# Supplementary figures and images for: MIC26 and MIC27 are bona fide subunits of the MICOS complex in mitochondria and do not exist as glycosylated apolipoproteins
Source: PLoS One. 2023 Jun 6;18(6):e0286756. doi: 10.1371/journal.pone.0286756 (PMC10243636; doi:10.1371/journal.pone.0286756)

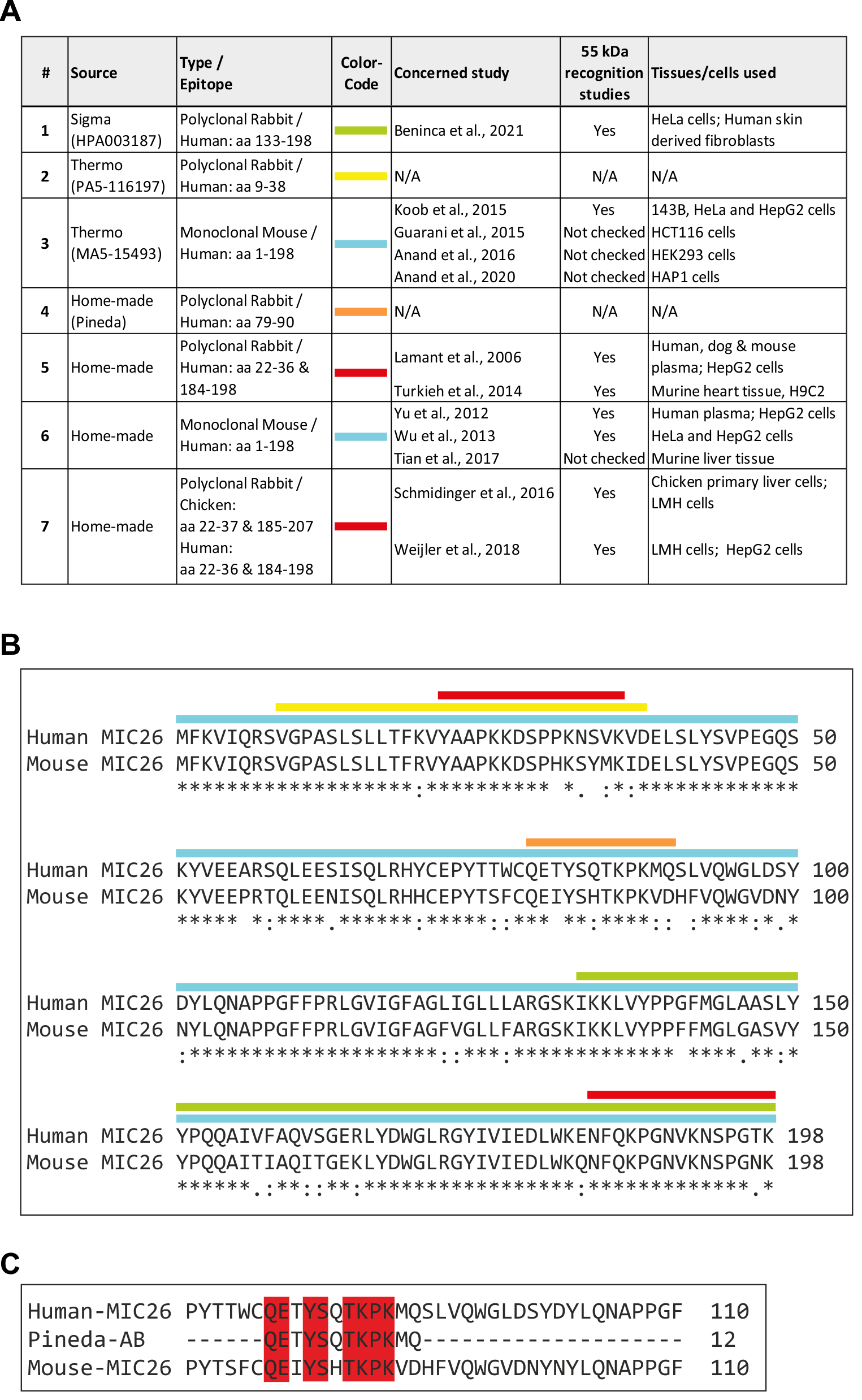

Supplement: S1 Fig — A) Summary of seven anti-MIC26 antibodies used in this study (#1-#4) and other (#1, #3, #5-#7) publications are described. The figure provides 1) the source of the antibody, 2) the host and region of the peptide used for antibody generation (amino acid numbers: aa), 3) the related color-code to (B), 4) publications describing the usage of the respective antibody, 5) if studies were performed regarding the 55 kDa form of MIC26 and if yes 6) which cell lines or tissues have been investigated. N/A is Not Applicable. B) Alignment of the human and mouse MIC26 amino acid sequence shows an identity of 83%. The epitopes used to generate different anti-MIC26 antibodies are highlighted by color-code and further described in the figure above. C) Alignment of the human and mouse MIC26 amino acid sequences, from position 61 to 110, with 12 amino-acid epitope used to generate antibody #4 (Home-made, Pineda) which shows proper alignment of antibody #4 epitope to human MIC26, but not mouse MIC26 where four mismatches were detected. Red color shows eight amino acids which are matching out of 12 amino acids. (TIF) [file pone.0286756.s002.tif]

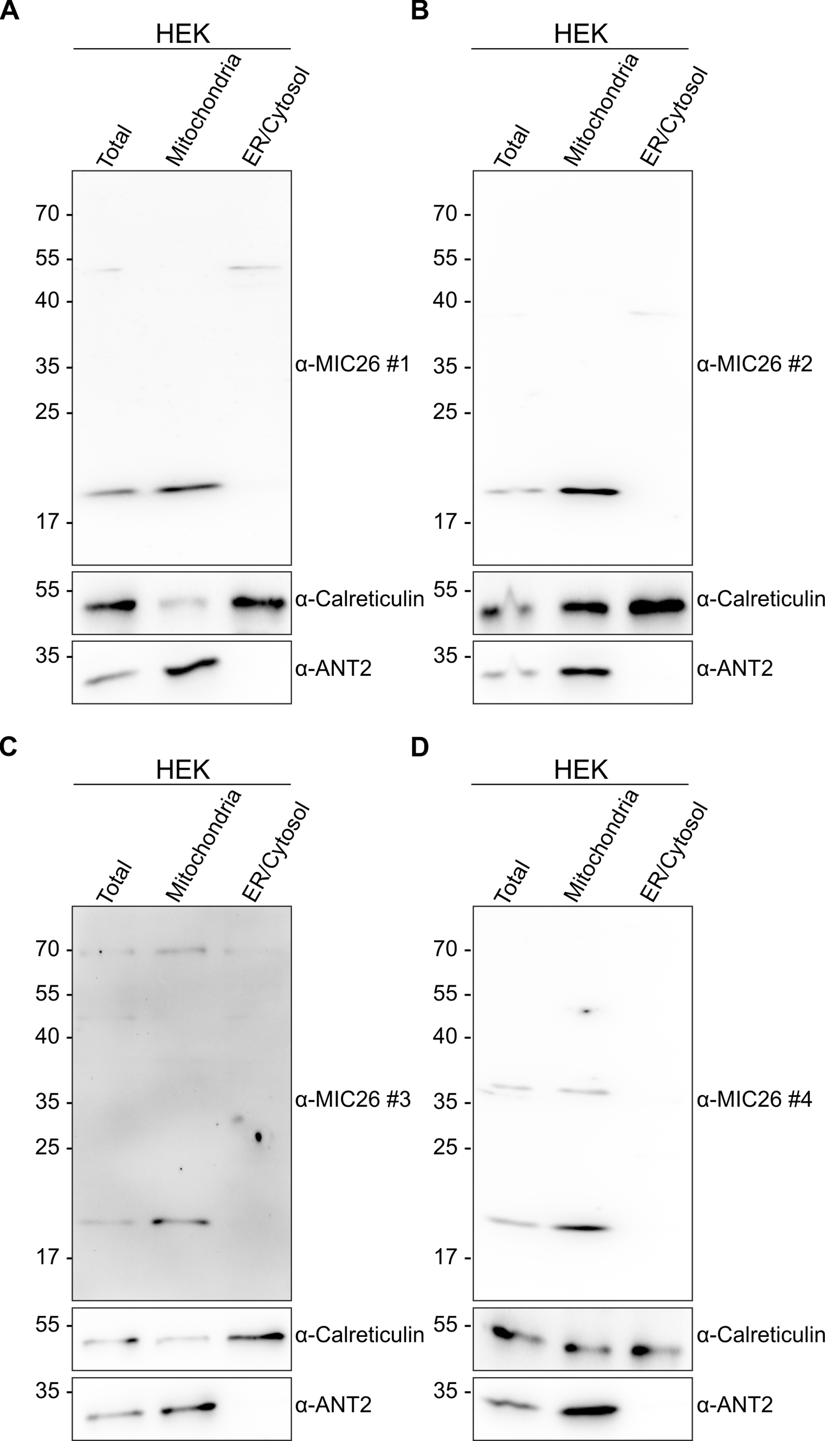

Supplement: S2 Fig — A) WBs showing the total cellular fraction, mitochondrial fraction as well as a fraction containing combined ER, Golgi and remaining cytosol, when anti-MIC26 antibody #1 was used. Antibodies against ANT2 and calreticulin served as markers for the mitochondrial as well as the combined ER, Golgi and cytosolic fraction respectively. B-D) WBs showing different fractions mentioned above where anti-MIC26 antibodies #2–4 were used. (TIF) [file pone.0286756.s003.tif]

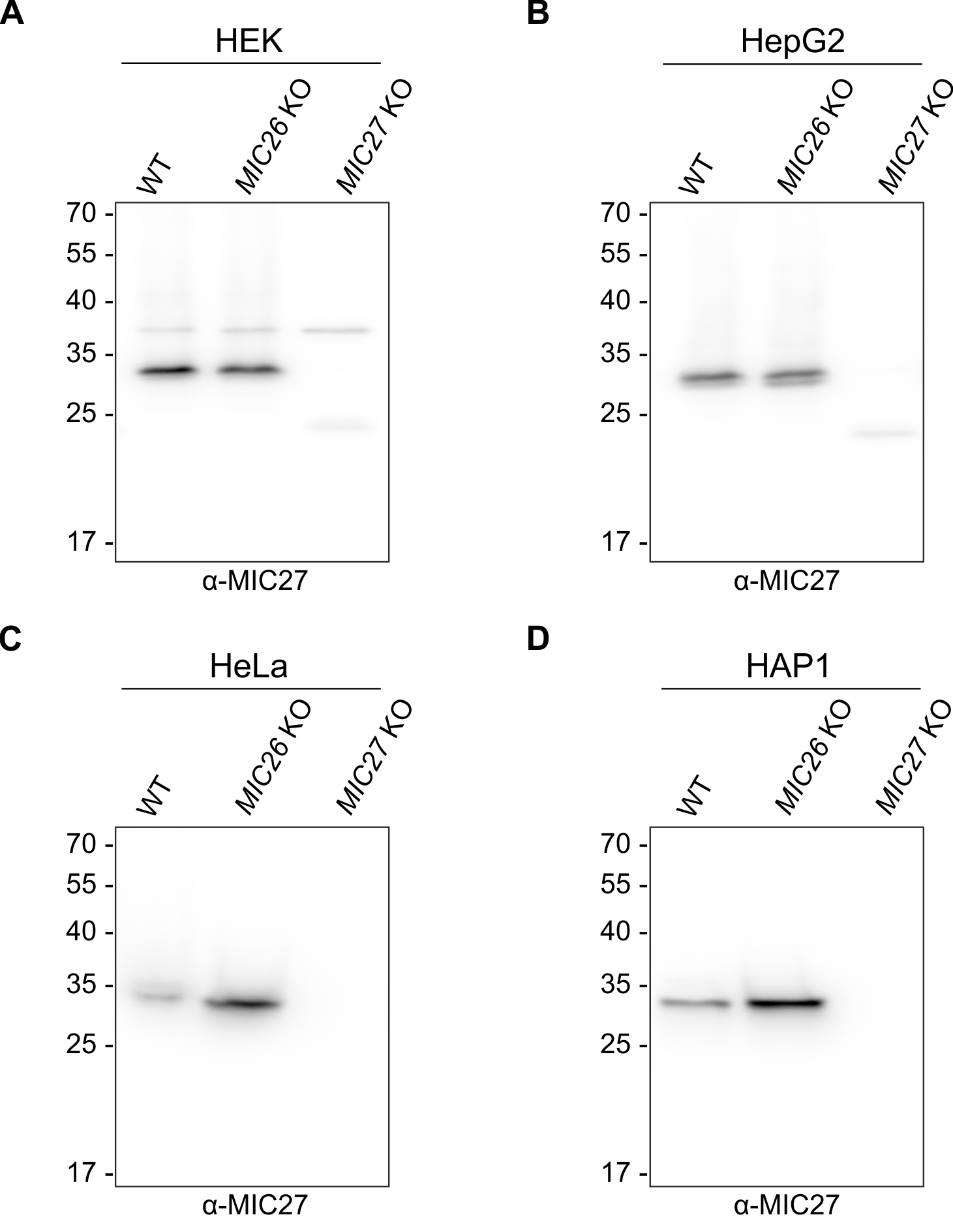

Supplement: S3 Fig — A-D) WB analysis of cell lysates from HEK293 (A), HepG2 (B), HeLa (C) and HAP1 (D) cells using anti-MIC27 antibody in WT, MIC26 KO and MIC27 KOs. Whole WBs are shown for clarity. (TIF) [file pone.0286756.s004.tif]

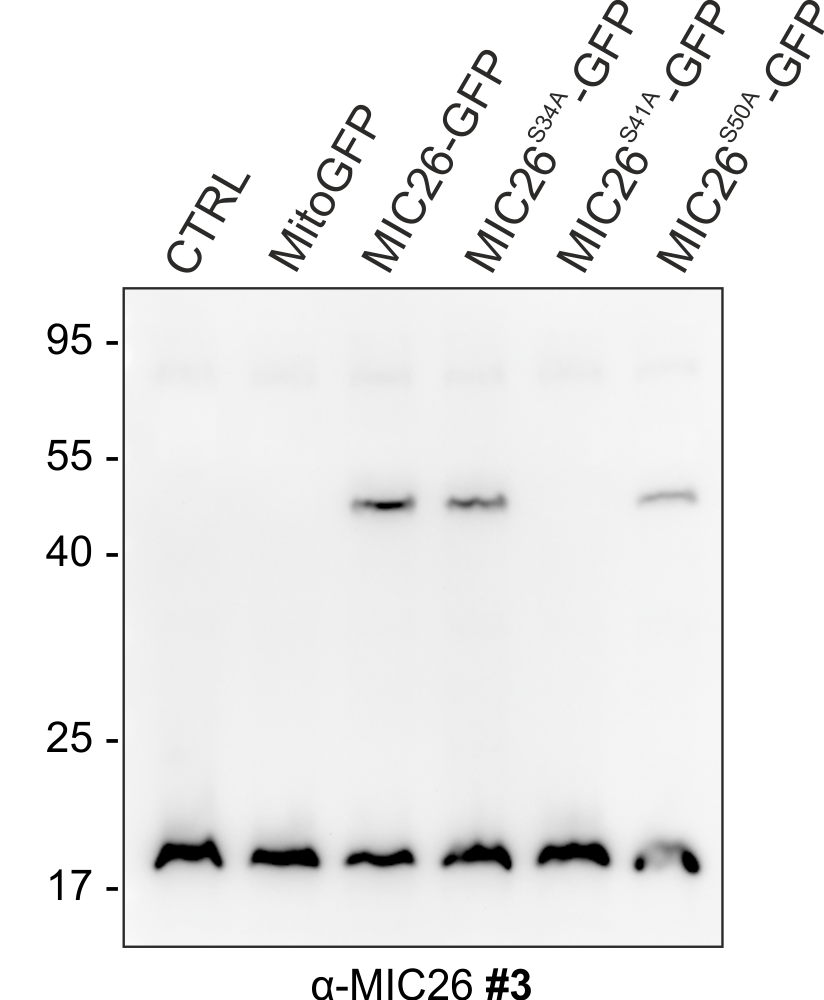

Supplement: S4 Fig — Antibody #3 detects the endogenous MIC2622kDa protein as well as MIC2622kDa-GFP. However, no MIC2622kDa-GFP protein was detected for the S41A mutant, leading to the assumption, that the antibody #3 has a strong binding affinity for serine in position 41. Furthermore, in accordance with anti-MIC26 antibody #1 and anti-GFP antibody (Fig 3), a ≈ 80 kDa MIC2655kDa-GFP protein was not recognized. (TIF) [file pone.0286756.s005.tif]

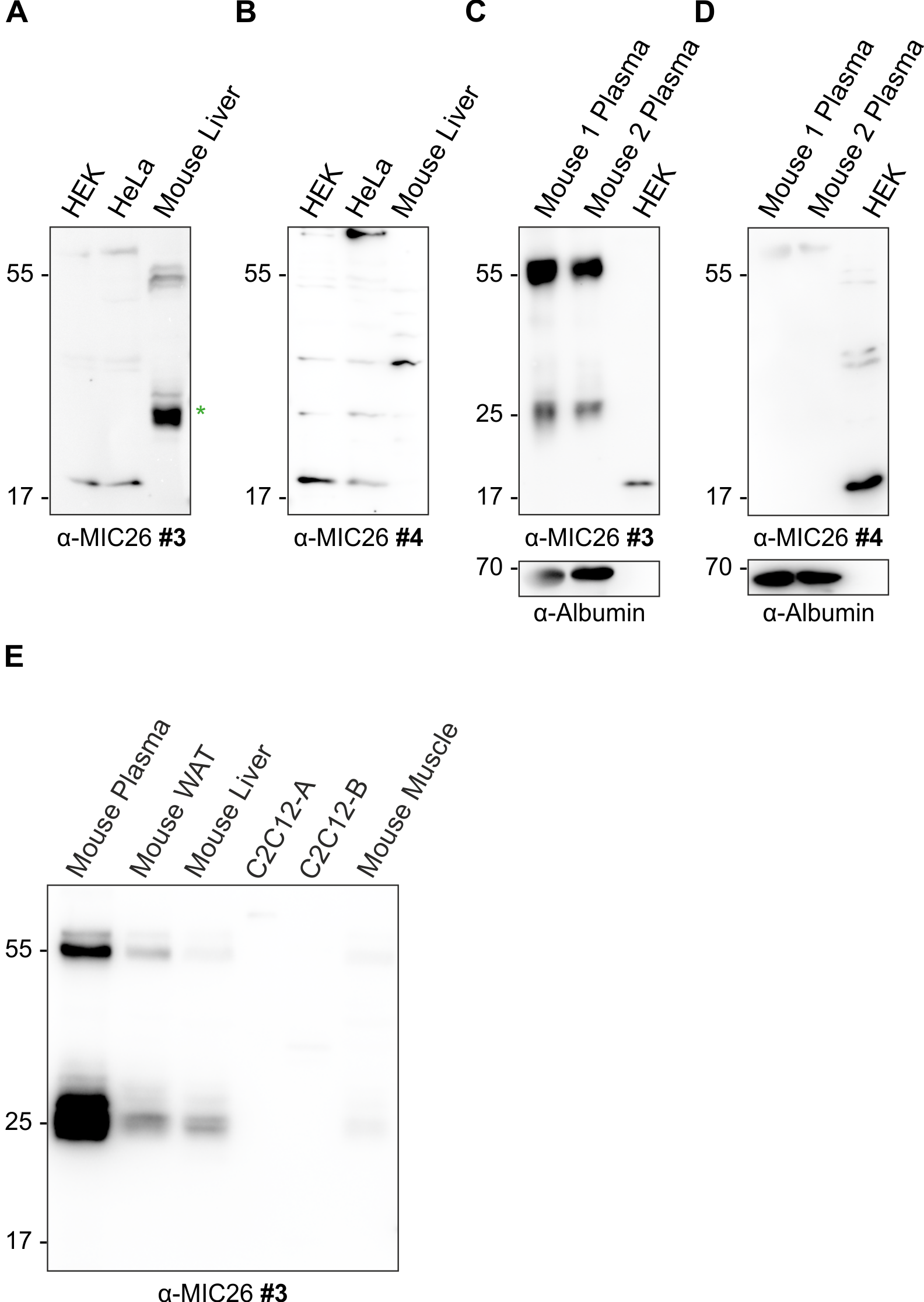

Supplement: S5 Fig — A) Antibody #3 shows immunoreactivity against MIC2622kDa in human cell lines but not murine liver tissue lysates, providing evidence regarding the unspecific nature of the detected 25 kDa and 55 kDa proteins in liver lysates. Additionally, antibody #3 shows an unspecific binding of ≈ 70 kDa protein in human cell lines. B) Antibody #4, comparable to antibody #3, shows immunoreactivity against MIC2622kDa in human cell lines but not murine liver tissue lysate revealing the unspecific nature of several additional bands detected in murine liver. C) Antibody #3 nonspecifically recognizes a 25 kDa and a 55 kDa protein in two different murine plasma samples, probably derived from light and heavy chain of IgG. D) Antibody #4 detects an unspecific band at approximately 70 kDa in murine plasma samples. E) Antibody #3 is not able to recognize MIC2622kDa protein in murine samples. However, it recognizes a 25 kDa and a 55 kDa band only in murine tissue samples but not in mouse cell lines indicating a nonspecific detection of the light and heavy chain of IgG. C2C12-A represents C2C12 cells lysed with RIPA buffer and C2C12-B represents C2C12 cells which were lysed mechanically. (TIF) [file pone.0286756.s006.tif]

Figure 1

**A**

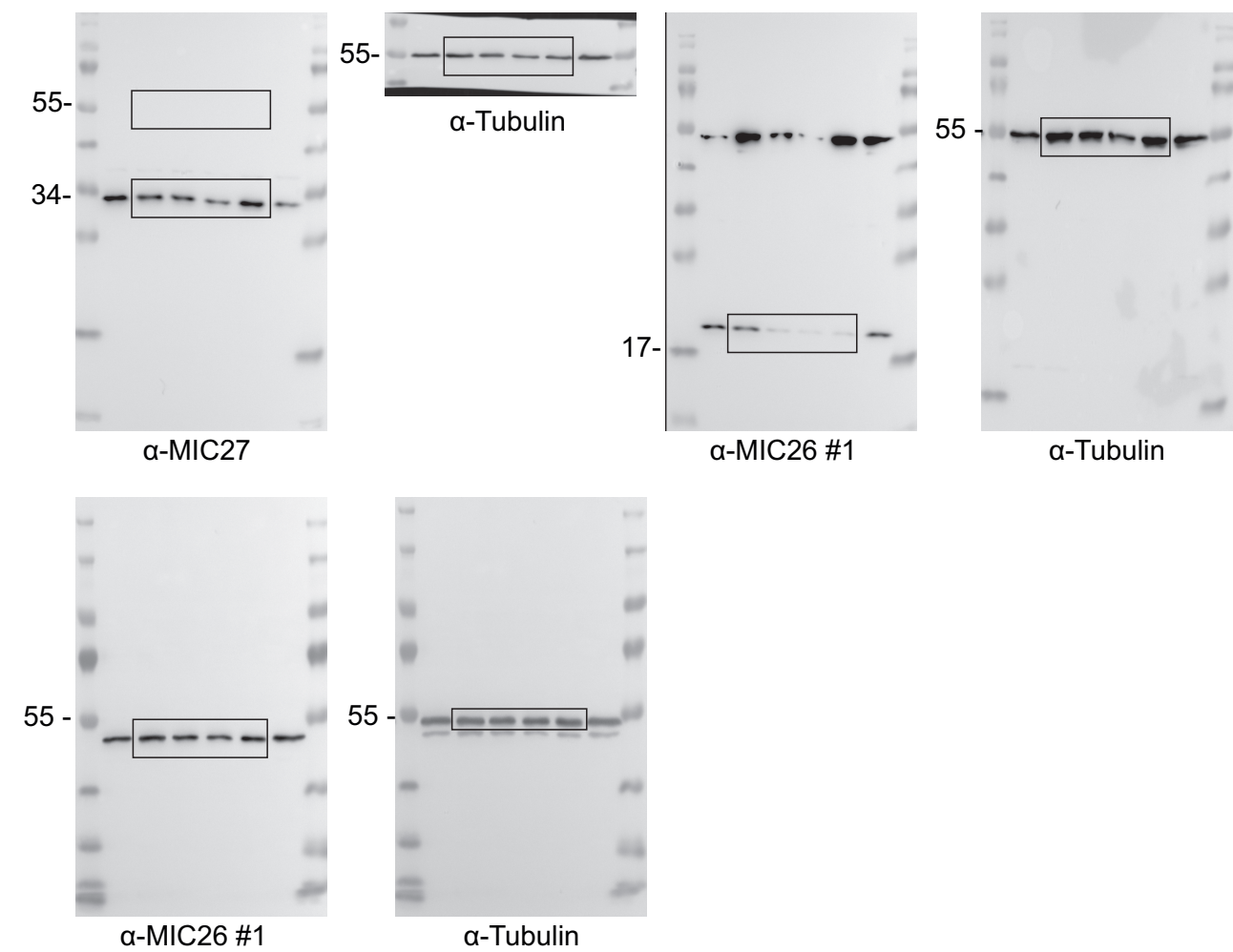

**B**

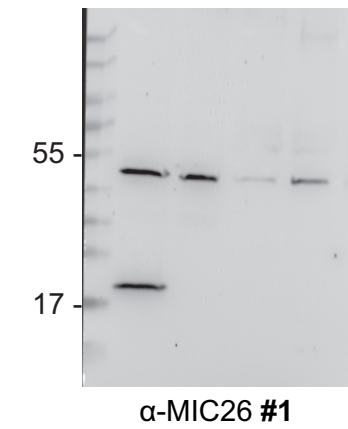

**C**

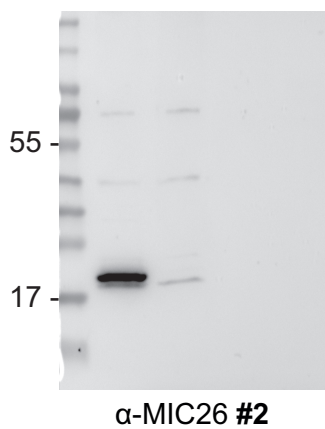

**D**

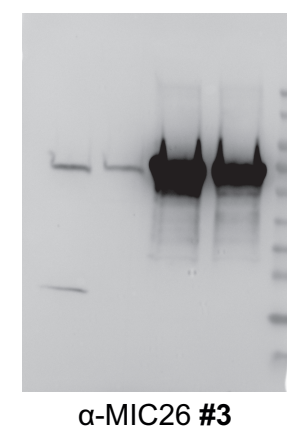

**E**

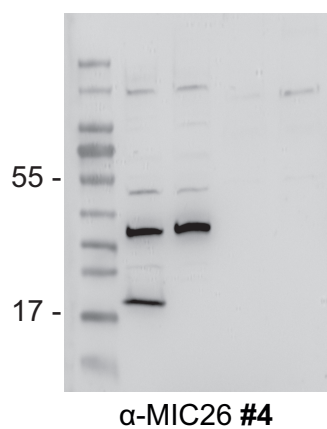

Figure 2

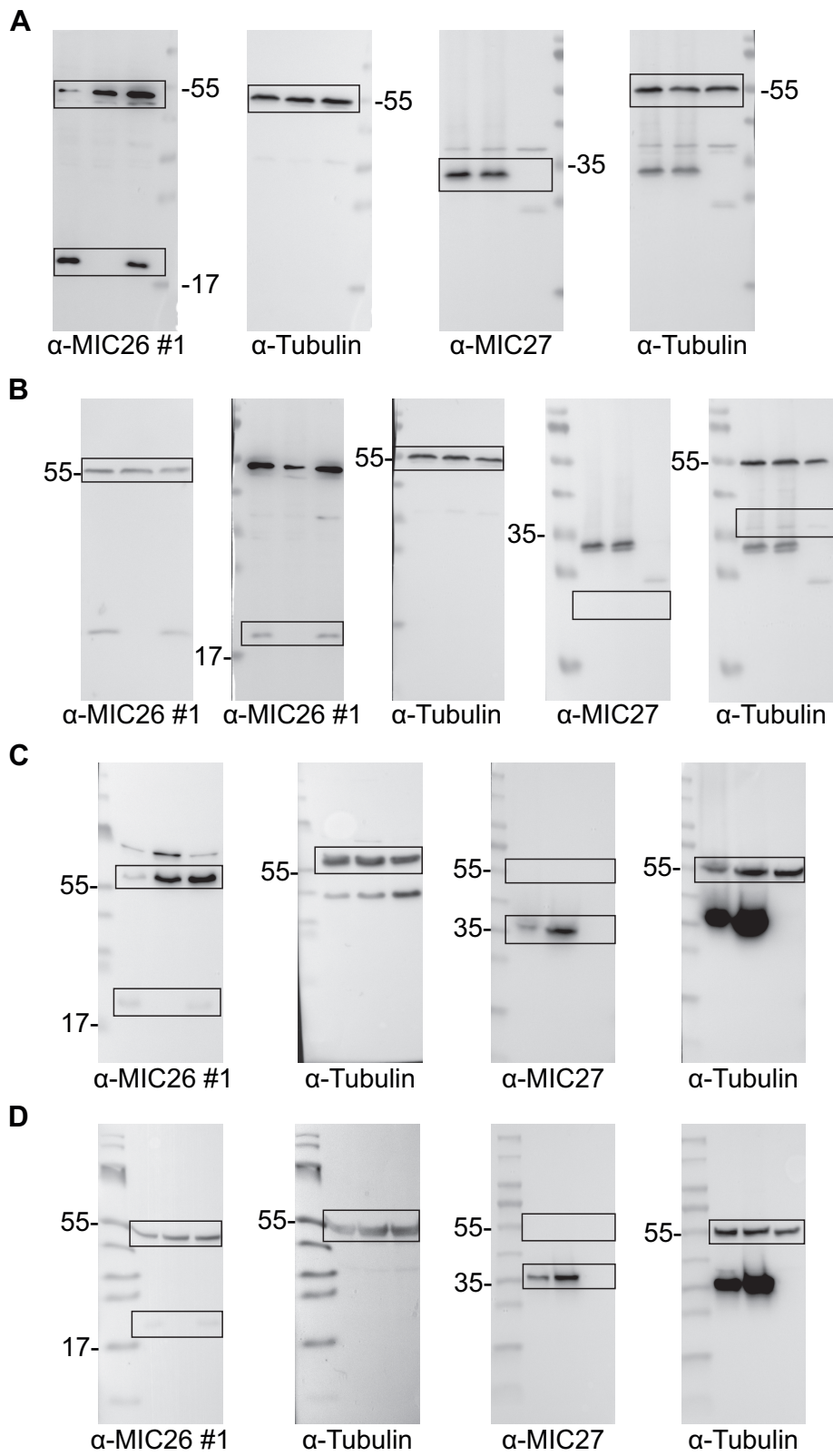

Figure 3

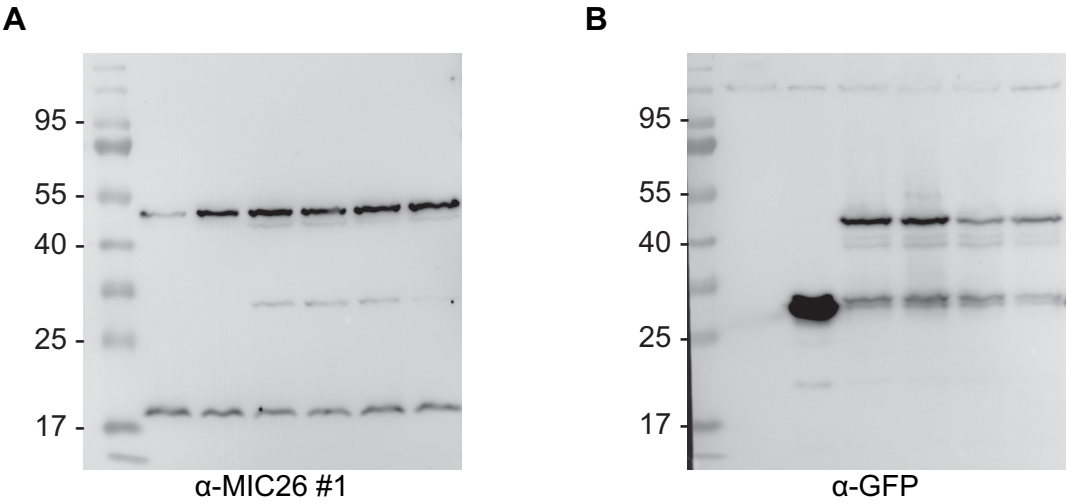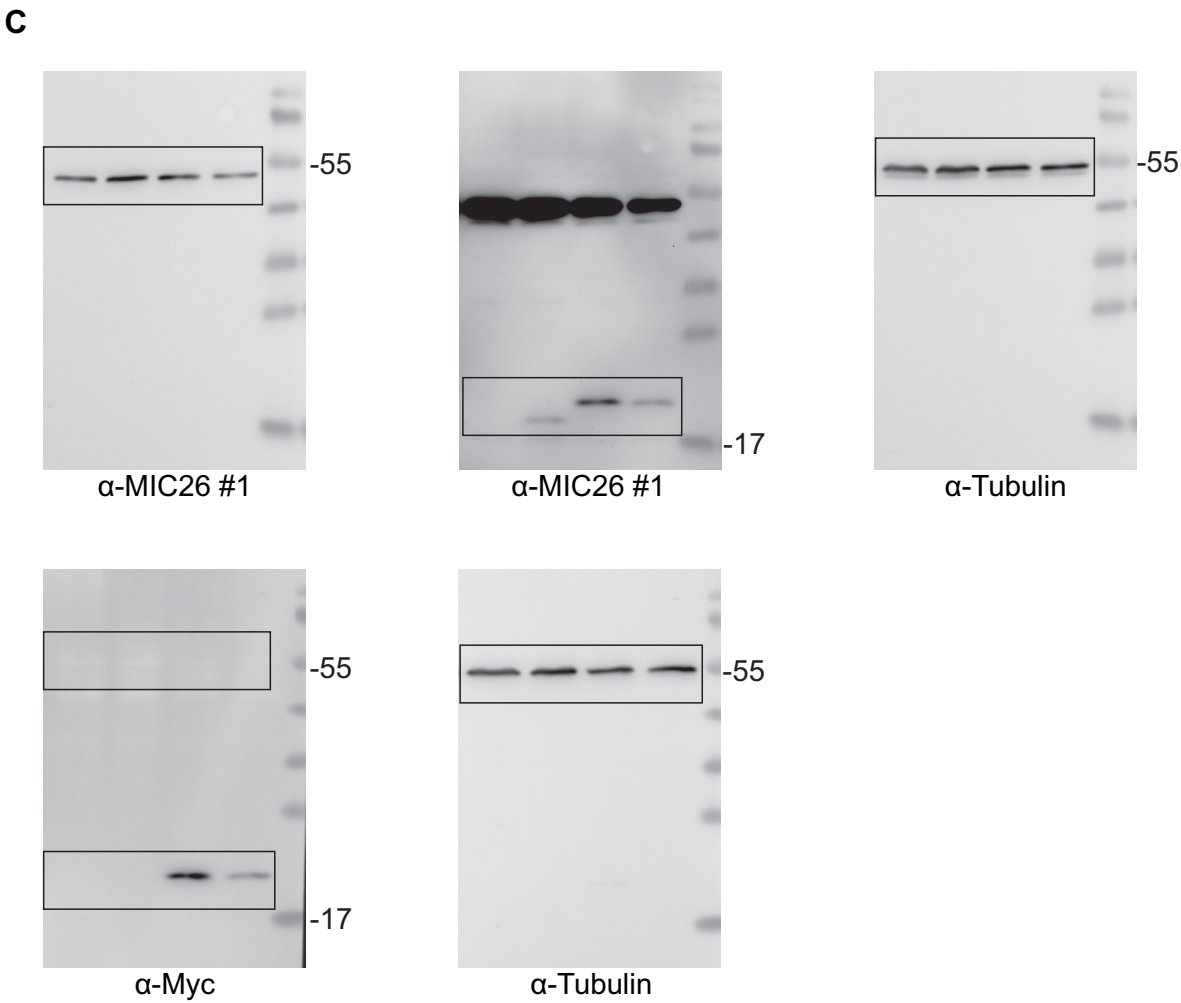

Figure 4

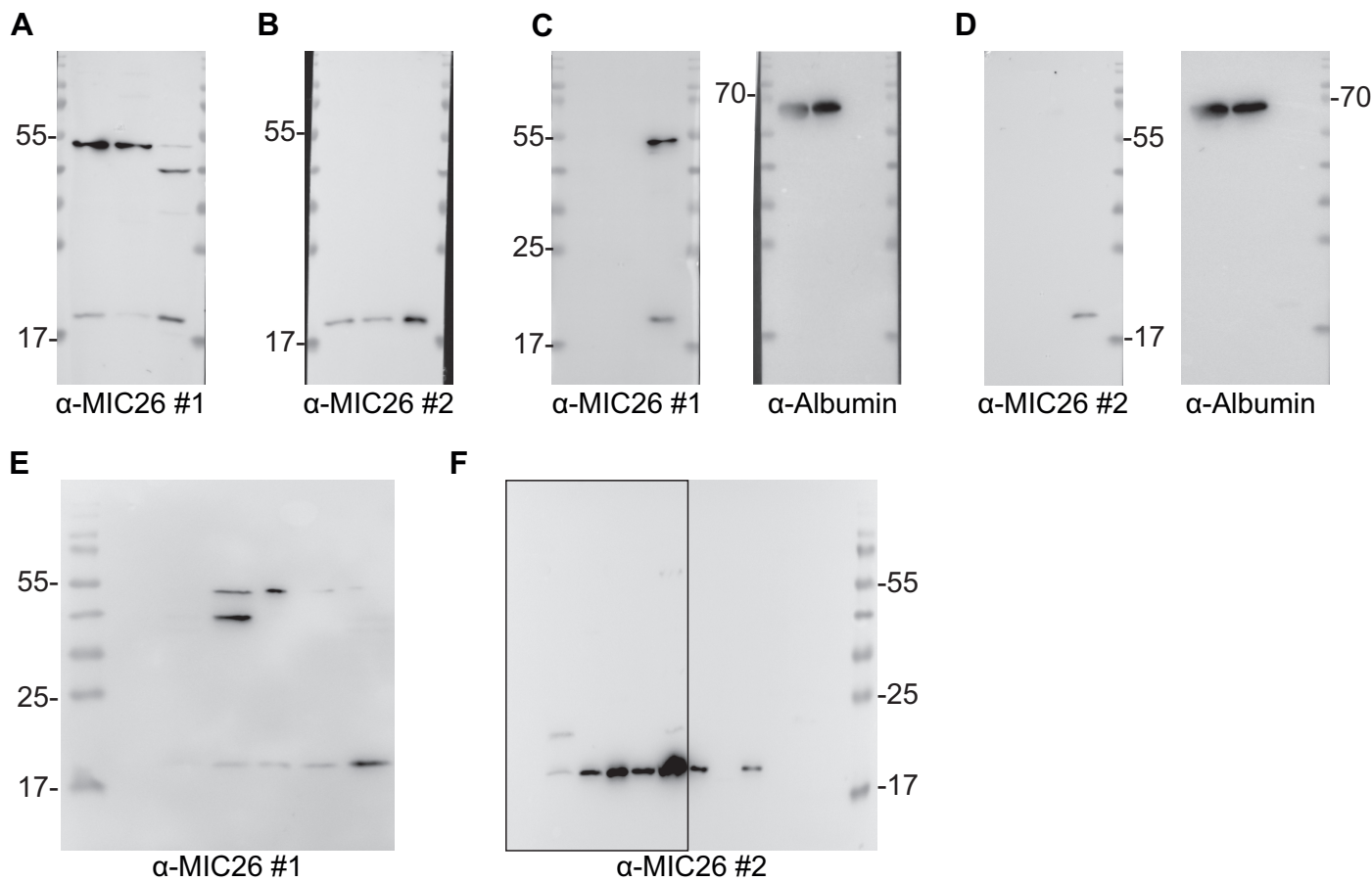

Figure 5

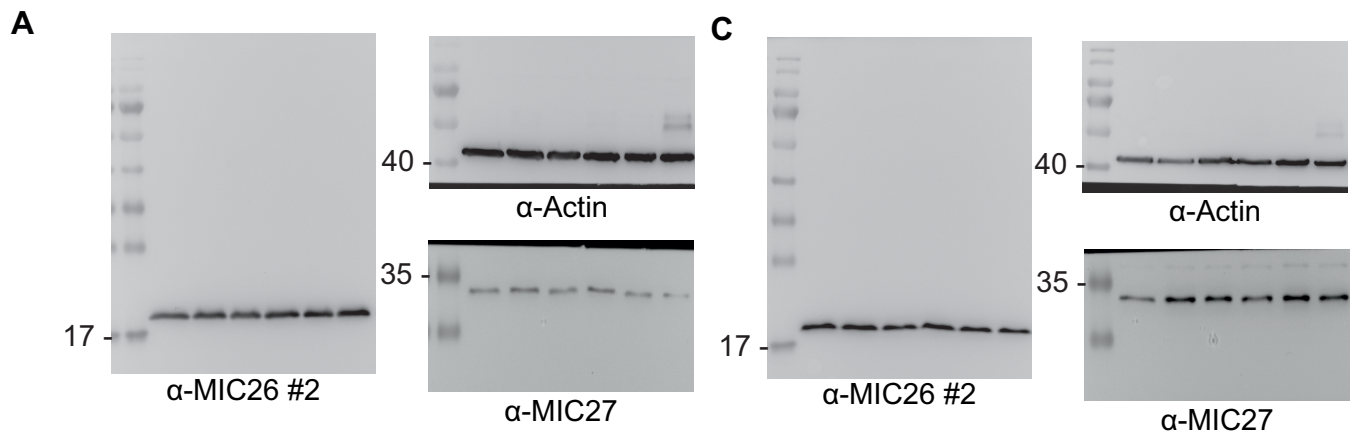

S2 Fig.

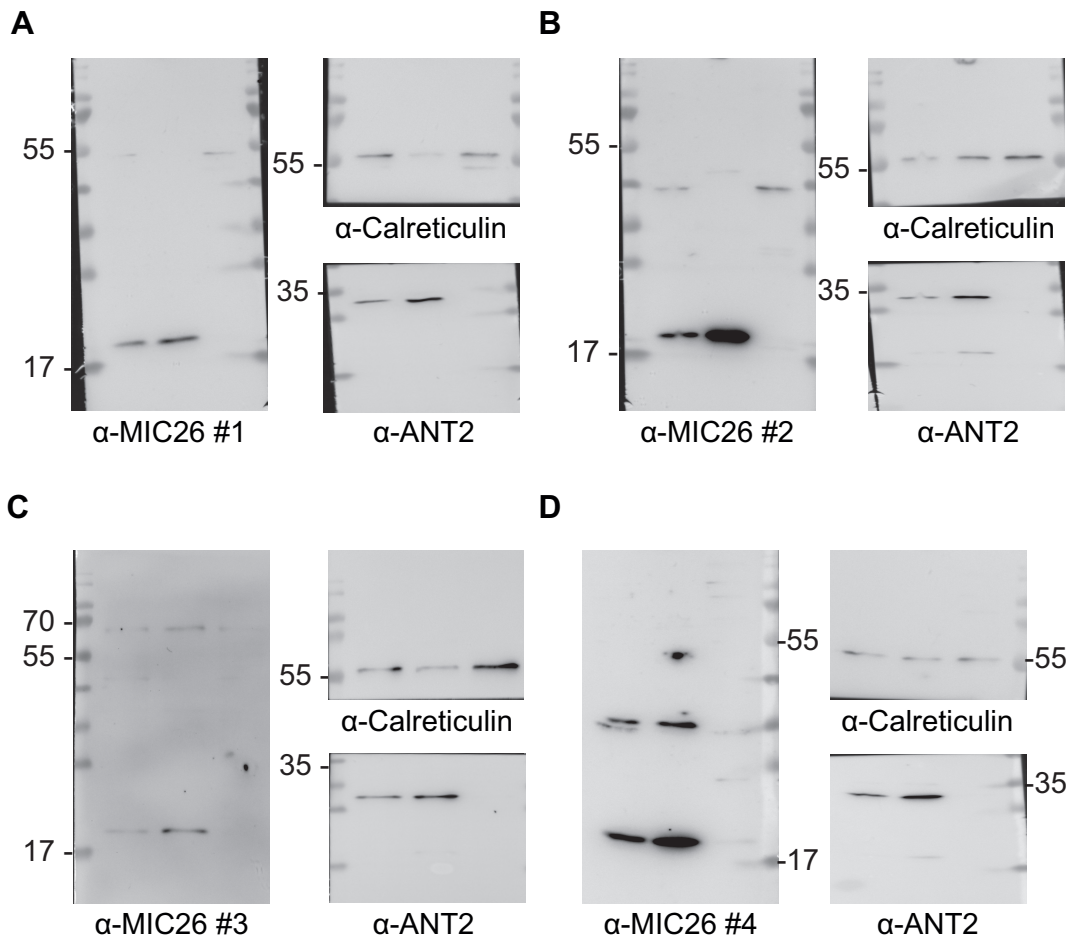

S4 Fig.

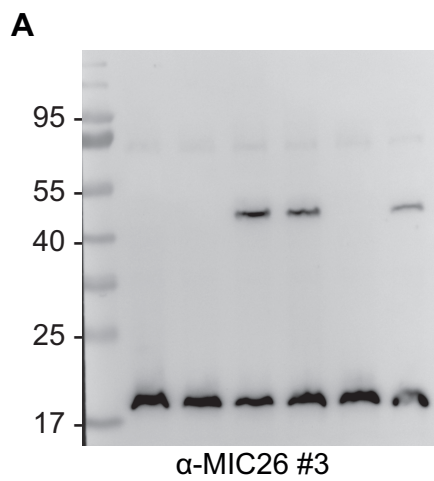

S5 Fig.

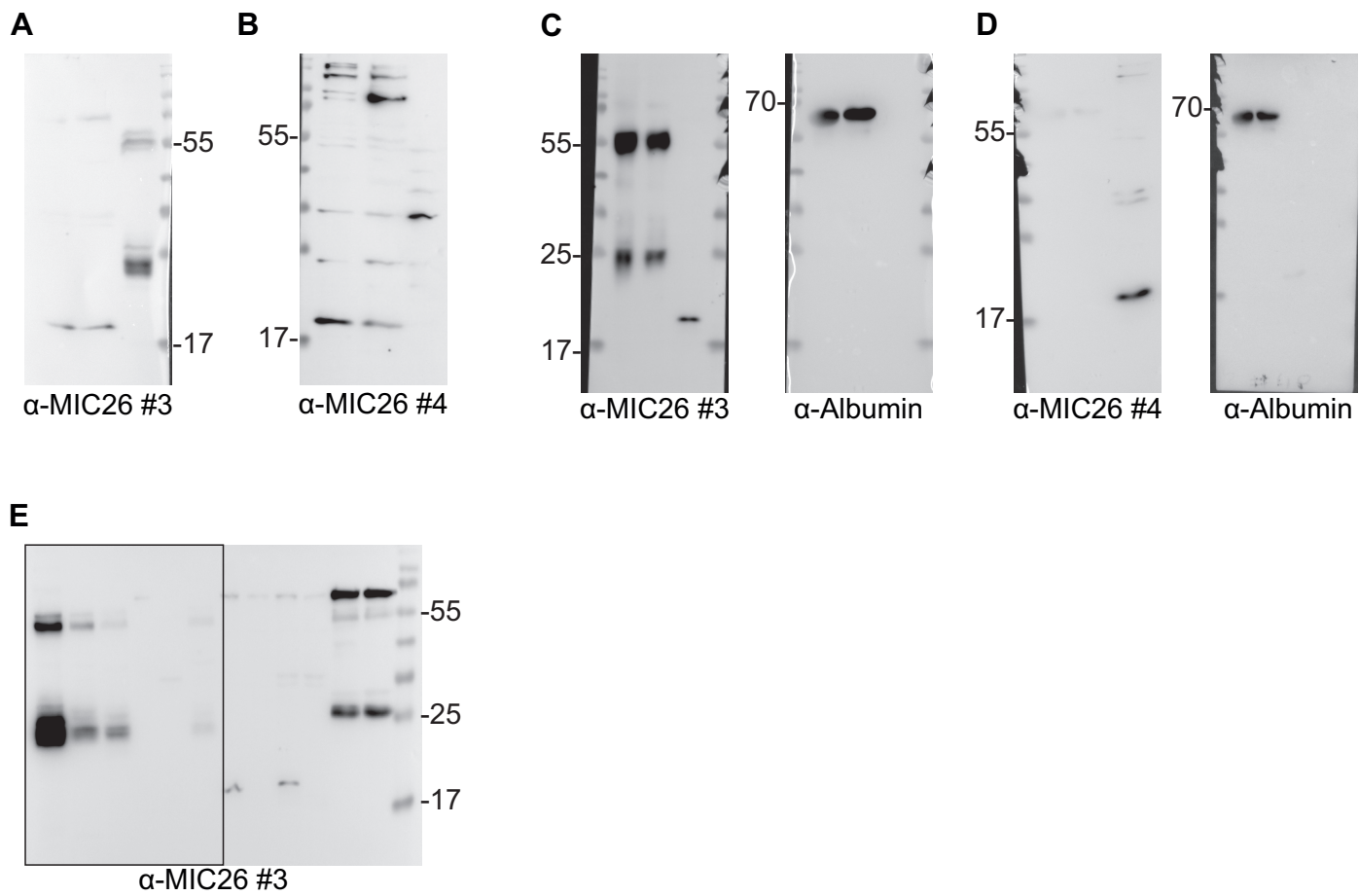

Supplement: S1 Raw images — (PDF) [file pone.0286756.s008.pdf]
